# lncRNAs–mRNAs Co–Expression Network Underlying Childhood B–Cell Acute Lymphoblastic Leukaemia: A Pilot Study

**DOI:** 10.3390/cancers12092489

**Published:** 2020-09-02

**Authors:** Ornella Affinito, Katia Pane, Giovanni Smaldone, Francesca Maria Orlandella, Peppino Mirabelli, Giuliana Beneduce, Rosanna Parasole, Mimmo Ripaldi, Marco Salvatore, Monica Franzese

**Affiliations:** 1 IRCCS SDN, Via E. Gianturco 113, 80143 Napoli, Italy; ornella.affinito@synlab.it (O.A.); katia.pane@synlab.it (K.P.); giovanni.smaldone@synlab.it (G.S.); francescamaria.orlandella@synlab.it (F.M.O.); direzionescientifica@sdn-napoli.it (M.S.); 2 Department of Paediatric Hematology–Oncology, Santobono-Pausilipon Hospital, 80143 Naples, Italy; beneduce_giuliana@libero.it (G.B.); rparasol64@gmail.com (R.P.); mimmo.ripaldi@tin.it (M.R.)

**Keywords:** RNA–Sequencing, long non–coding RNA, leukaemia, diagnostic, bioinformatics, biomarker, NGS, network, co–expression, feature extraction

## Abstract

**Simple Summary:**

Acute lymphoblastic leukemia (ALL) is one of the most common childhood cancers. The ALL onset involves abnormal proliferation and arrest of differentiation of B or T cell progenitors. Recently, long non–coding RNAs (lncRNAs) gained great interest in the B–ALL leukemogenesis, however, so far few “omic” studies investigate lncRNAs and protein–coding gene networks. In our retrospective study, we conceived an integrated bioinformatic approach, by using NGS platform, to discover lncRNAs strongly correlated with aberrantly expressed protein–coding genes. We provided dysregulated lncRNA–mRNA pairs potentially underlying B–ALL pathogenesis. Diagnosis incidence peak of ALL appears approximatively between 1 and 19 years old. lncRNAs may be of clinical utility as non–invasive biomarker for B–ALL onset or therapy response in support of precision medicine. The identification of lncRNA as key regulators in B–ALL could lead to the identification of the altered pathways able to sustain the leukemic growth.

**Abstract:**

Long non–coding RNAs (lncRNAs) are emerging as key gene regulators in the pathogenesis and development of various cancers including B lymphoblastic leukaemia (B–ALL). In this pilot study, we used RNA–Seq transcriptomic data for identifying novel lncRNA–mRNA cooperative pairs involved in childhood B–ALL pathogenesis. We conceived a bioinformatic pipeline based on unsupervised PCA feature extraction approach and stringent statistical criteria to extract potential childhood B–ALL lncRNA signatures. We then constructed a co–expression network of the aberrantly expressed lncRNAs (30) and protein–coding genes (754). We cross–validated our in–silico findings on an independent dataset and assessed the expression levels of the most differentially expressed lncRNAs and their co–expressed mRNAs through ex vivo experiments. Using the guilt–by–association approach, we predicted lncRNA functions based on their perfectly co–expressed mRNAs (Spearman’s correlation) that resulted closely disease–associated. We shed light on 24 key lncRNAs and their co–expressed mRNAs which may play an important role in B–ALL pathogenesis. Our results may be of clinical utility for diagnostic and/or prognostic purposes in paediatric B–ALL management.

## 1. Introduction

Acute lymphoblastic leukaemia (ALL) is the most common childhood blood cancer [1,2] and it is due to the malignant transformation and consequent uncontrolled proliferation of B or T cell precursors normally residing in the bone marrow [1,3]. B lymphoblastic leukaemia (B–ALL) is characterized by different oncogenic alterations at cytogenetic and molecular level [1,3].With the advent of next–generation sequencing (NGS) technologies, remarkable progress has been made in the understanding of the molecular basis of B–ALL [4]. Concomitantly, novel expression signatures have emerged in leukemogenesis for diagnostic and therapeutic purposes, including long non–coding RNAs (lncRNAs) [5,6]. lncRNAs are 200 nucleotides long RNA molecules with pivotal regulatory roles such as chromatin structure remodelling, epigenetics, transcriptional and post–transcriptional processing [7,8]. Increasing studies highlight that lncRNAs may contribute to tumourigenesis and have the potential for diagnostic and therapeutic applications [9,10,11,12,13,14].

Several aberrantly expressed lncRNAs have been found associated with B–ALL [15,16,17,18,19,20,21,22]. For instance, Fernando et al. [19] highlighted the over–expression of the lncRNA CASC15 with regulatory roles on the transcriptional factor SOX4, in paediatric RUNX–1 translocated B–ALL. Interestingly, Lajoie et al. [20] identified a list of lncRNAs that similarly enable B–ALL subtype stratification as for protein–coding genes. Moreover, lncRNAs BALR–2 and BALR–6 have tumour–suppressor potential promoting cell survival in B–ALL and cell lines [18]. Another lncRNA, RP11–137H2.4 possesses tumour suppressor activity; indeed, in vitro in human pre–B–cell lines, silencing, has a strong impact on cell proliferation, migration and apoptosis [15].

High–throughput RNA sequencing (RNA–Seq) technology enables the identification of functional long non–coding RNAs [23]. Moreover, in order to face the high RNA–Seq data dimensionality, a notable unsupervised dimension reduction method, is the Principal Component Analysis (PCA) [24,25,26,27], which selected few significant features without loss of biological information.

Although many lncRNAs have been genetically mapped into the genome, their functions and regulatory mechanisms are still under investigation [7]. The computational prediction of lncRNA functions may rely on lncRNA–mRNA co–expression networks and “guilt–by–association” strategy [28,29,30]. The idea is that functionally annotated and unknown genes within a co–expressed module may cooperate to exert similar functions, allowing to associate unknown gene functions [29,30]. Several co–expression network approaches have been successfully applied for studying different cancers [31,32].

Recently, our group highlighted KCTD15, a Potassium Channel Tetramerization Domain Containing 15 protein, over–expressed in childhood B–ALL patients compared with healthy subject samples through RNA–Sequencing experiments (RNA–Seq) (Bioproject: PRJNA601326) [33].

In this pilot study, we aim to identify novel lncRNA–mRNA pairs involved in B–ALL pathogenesis. We applied a PCA–based unsupervised feature extraction (FE) approach to extract candidate lncRNA signatures distinctive of B–ALL patients over the healthy subjects. Next, we constructed a co–expression network of the aberrantly expressed lncRNAs and protein–coding genes (PCGs). We assessed our in–silico findings by RNA–Seq independent childhood B–ALL dataset and ex vivo Quantitative reverse transcription PCR (RT–qPCR) experiments on our internal cohort. Using the guilt–by–association approach, we unravel 24 key lncRNAs and their perfectly co–expressed mRNAs potentially underlying B–ALL. Our results suggest that several lncRNAs, and the corresponding co–expressed mRNAs, may play an important role in cancerous signalling pathways and may be of clinical utility for diagnostic and/or prognostic purposes.

## 2. Results

**Figure 1:**
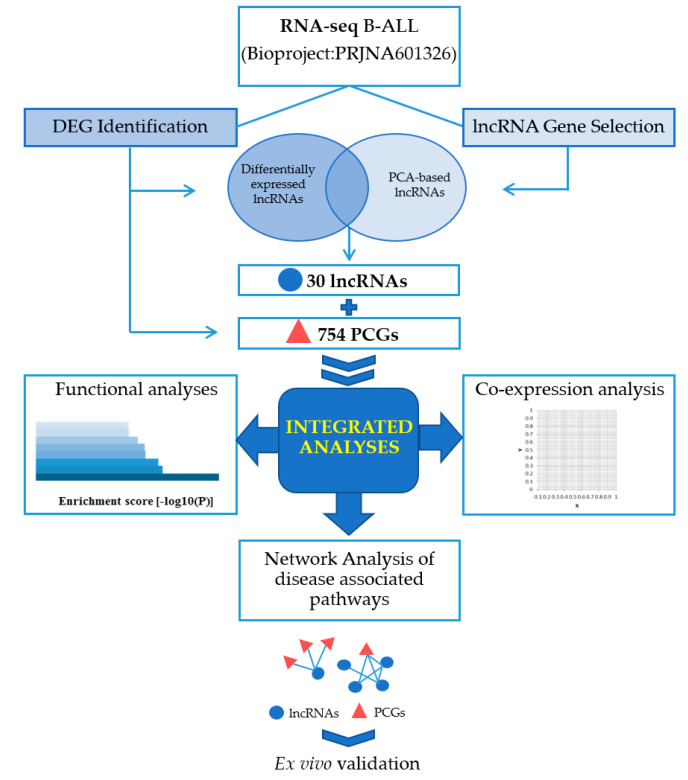
Methodological study workflow. Abbreviations: long non-coding RNAs (lncRNAs), differentially expressed genes (DEGs), protein-coding genes (PCGs), principal component analysis (PCA).

The methodological workflow for this study is summarized in Figure 1.

### 2.1. The Landscape of B–ALL Transcriptome

Transcriptomic analysis from 6 samples (3 healthy subjects and 3 B–ALL patients) allowed us to quantify the expression of 17,085 genes. Raw counts for these genes were normalized by using the variance stabilizing transformation (vst) to obtain a matrix of homoscedastic values as shown before and after normalization (Figure S1A,B, respectively). The distribution of expression levels in healthy subjects and B–ALL patients is reported in Figure S1C. Only genes (16319), whose biotype was available in Ensembl GRCh37.p13, were retained for further analysis. Among 16,319 genes, 73% represented protein–coding transcripts, 13.6% were assigned to long and 3.1% to short non–coding transcript types, 9.8% were identified as pseudogenes and 0.37% represented other biotype categories (immunoglobulin genes, T cell receptor genes and mitochondrial genes) (Figure S1D). Among lncRNAs, 5.9% were annotated as antisense, 4.7% as long intergenic RNAs, 0.9% belonged to processed transcripts, 1.7% were sense–intronic RNAs, 0.36% were sense–overlapping RNAs and 0.02% were 3′ overlapping ncRNAs.

### 2.2. Identification of Key lncRNAs in B–ALL through Unsupervised PCA–Based Feature Extraction (FE) Approach

In order to identify lncRNAs able to differentiate healthy from tumour patients, we performed a principal component analysis (PCA) based on unsupervised feature extraction (FE) analysis. PCA reduces the dimensionality of the data while retaining most of the variation in the predictor variables. Our dataset is composed of 2016 lncRNAs genes. We applied the PCA on lncRNAs expression profiles as a multivariate framework to reduce the number of features (lncRNAs) and to extract the most informative lncRNAs able to characterize B–ALL disease.

**Figure 2:**
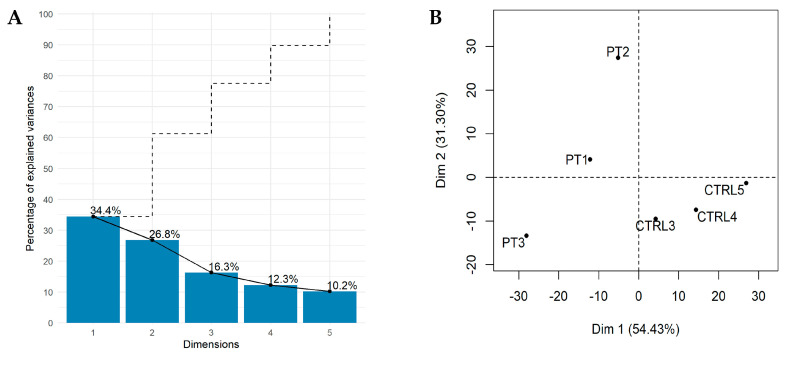
PCA–based feature extraction analysis. In Scree diagram plot (**A**) the proportion of variance and cumulative variance of principal components are shown. Each bar represents an eigenvector (principal component); the numbers above each bar represents the eigenvalue (variance explained) of each eigenvector. The dashed line represents the cumulative explained variance. In (**B**) samples are plotted by using the first two principal components (PC1 and PC2). In the brackets, the variance explained by each principal component.

To reduce the dimensionality of our dataset, we only selected the principal components (PCs) that contain most of the information, maximizing data variance. Figure 2A shows the variance explained by each component and the corresponding cumulative explained variance.

The cumulative variance analysis shows that most of the variance is contained in the first two PCs (about 60%) (Figure 2A). Thus, the most significantly correlated variables (lncRNAs) (Pearson correlation; FDR ≤ 0.05) with each one of the two PCs were extracted. Specifically, we extracted a total of 584 lncRNAs: out of these, 379 were uniquely associated with the first PC and 205 with the second PC. We finally performed the PCA using only these 584 lncRNAs to confirm that they discriminate healthy subjects from B–ALL patients (Figure 2B). It is clear that the tumour patients and healthy subjects were well separated in the two–dimensional space spanned by the PC1 and PC2 loadings. This suggests that the unsupervised PCA–based FE successfully identified a limited and low number of lncRNAs genes that well discriminate between the two groups capturing high variability data. Furthermore, our PCA–based approach filtered lncRNAs proportionally distributed in different quartiles. Notably, these lncRNAs fall within the low, medium and high differential expression levels (Figure S2).

### 2.3. lncRNA Signature as Diagnostic Candidate for B–ALL

The intersection of the PCA–based 584 lncRNAs with the 873 differentially expressed genes (DEGs) from the transcriptome dataset [33] resulted in 30 differentially expressed lncRNAs (DElncRNAs, Table 1), hereinafter referred only as candidate lncRNAs

Hierarchical clustering was used to analyse the expression profiles of the 30 lncRNAs (Figure S3). Out of these, 24 lncRNAs were down–regulated and 6 lncRNAs were up–regulated. Of the dysregulated lncRNAs, 20 lncRNAs changed with a fold change (FC) of more than 3 compared with healthy subjects (Table 1). The most up–regulated lncRNA was *TEX41* (ENSG00000226674), with a fold change (FC) of more than 5, and the most down–regulated lncRNAs were *RP11_534L6.2* and *SNX29P2* (ENSG00000236800 and ENSG00000198106, respectively) with a FC of −4.3 and −4.2, respectively.

To the best of our knowledge, these three lncRNAs (*TEX41, RP11_534L62* and *SNX29P2*) have never been associated with the B–ALL. Indeed, from literature, *TEX41* has been involved in human tumours such as squamous cell carcinoma based on a TCGA cohort [42] and recently emerged among novel aortic valve stenosis disease loci [43]. The *RP11_534L6.2* and *SNX29P2* are still uncharacterized as reported in Table 1.

**Figure 3:**
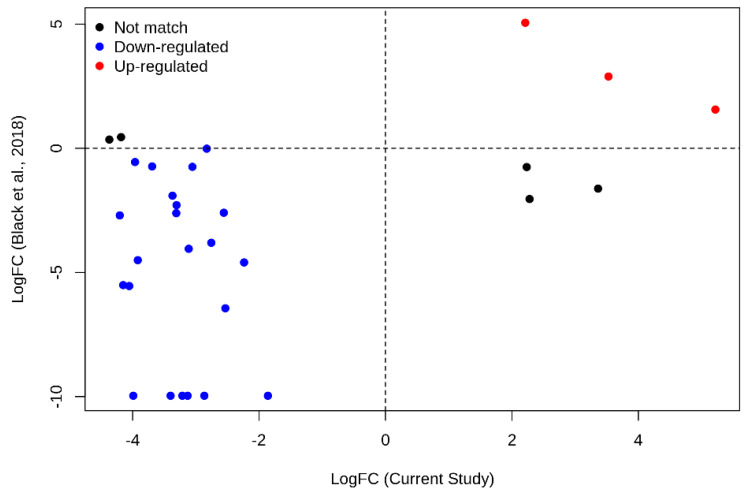
In–silico validation using PRJNA475681. Scatter plot showing the expression trend (i.e., logFC) of the 30 lncRNAs found in the current study compared with the Bioproject PRJNA475681 (childhood B-ALL RNA-Seq dataset by Black et al. [50], 2018). The values relative to lncRNAs scored down-regulated and up-regulated in both studies are highlighted in blue and red, respectively; instead, values relative to lncRNA with the opposite trend in the two studies are highlighted in black.

The expression trend of the 30 differential lncRNAs herein identified, has been compared with a public childhood B–ALL RNA–Seq dataset (Accession Number PRJNA475681, by Black et al., 2018 [50]). We evaluated the expression changes (i.e., log_2_FC) of three B–ALL patients with t(12;21) (ETV6/RUNX1) and healthy subjects. Notably, most lncRNAs (n.25 out of 30) showed expression trend concordance with the Black et al. [50] dataset as shown in Figure 3 (blue and red lncRNAs, down–regulated and up–regulated, respectively). Only 5 genes show an opposite trend.

We evaluated the statistical significance associated to the proportion of concordant lncRNAs in term of expression by Binomial Test with a probability of success equal to 0.61, corresponding to the proportion of 7997 differentially expressed lncRNAs (5653 down–regulated and 2344 up–regulated) respected to 13,144 total lncRNAs in the Black’s dataset (*p*–value = 0.007645).

### 2.4. Functional Analyses and Co–Expression Network

#### 2.4.1. Signalling Pathways of Differentially Expressed mRNAs and lncRNAs

**Figure 4:**
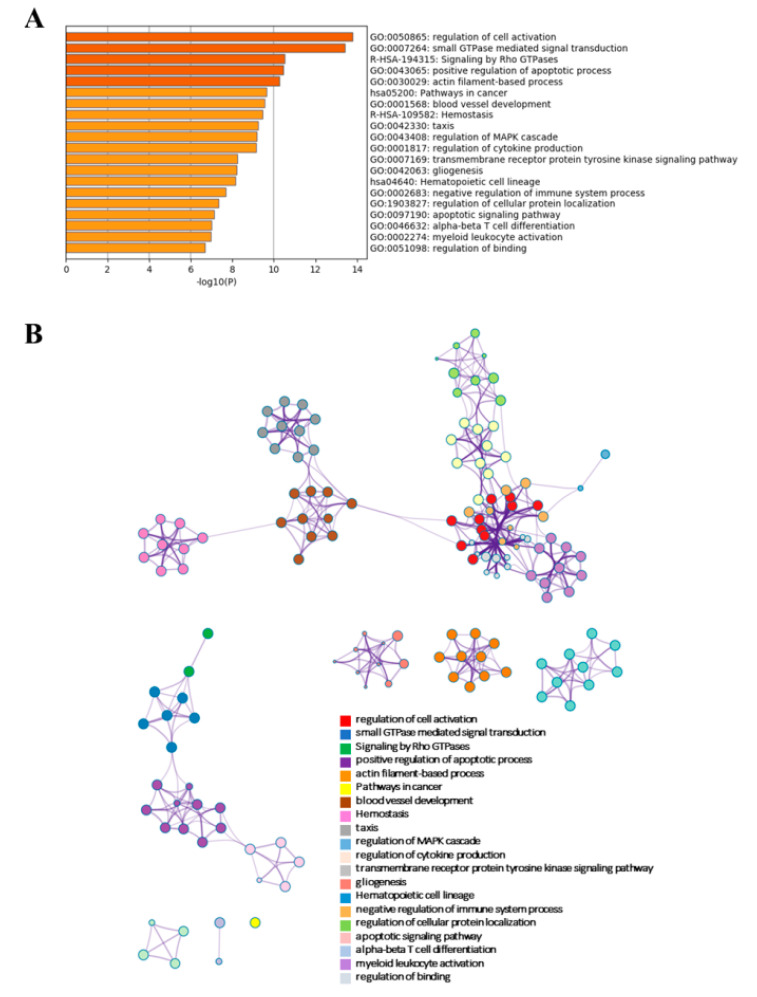
Signalling pathways regulated by protein–coding genes (PCGs) according to Gene Ontology (GO), Reactome and Kyoto Encyclopedia of Genes and Genomes (KEGG) database. (**A**) Top statistically significant enriched signalling pathways (−log10*p* > 6) with accumulative hypergeometric *p*–values and enrichment factors calculated and used for filtering from overall enrichment analysis. (**B**) Enriched ontology clusters shown as a network only for the subset of representative biological processes (−log10*p* > 6, accumulative hypergeometric *p*–values) from the full enrichment analysis. Each term is represented by a circle node. Top statistically significant terms are hierarchically clustered into a tree based on Kappa–statistical similarities (score > 0.3) among their gene memberships, linked by an edge (the thickness of the edge represents the similarity score). Each cluster has a colour and term description.

As mentioned above, the PCA, combined with differential expression analysis, prioritized 30 lncRNAs able to distinguish B–ALL patients from healthy subjects. To predict lncRNAs function, we firstly carried out functional enrichment analyses using the protein–coding genes (PCGs) differentially expressed in B–ALL relative to normal samples from RNA–Seq data [33] hereinafter referred as PCGs. Figure 4A results showed the top 20 clusters of the most statistically significant enriched terms from multiple ontologies, i.e., gene ontology (GO), Kyoto Encyclopedia of Genes and Genomes (KEGG) and Reactome database ranked for statistical significance.

PCGs were primarily involved in clusters related to the “Regulation of cell activation (GO:0050865)” including lymphocyte proliferation and differentiation terms; “small GTPase signalling transduction”, “ actin filament–based process (GO:0030029)” and “Signalling by Rho GTPase (R–HSA–194315)” including the cell morphology and/or cytoskeleton organization terms. Moreover, PCGs regulated typical cancer signalling hallmarks such as “transmembrane receptor protein tyrosine kinase signalling pathway (GO:0007169)” and “positive regulation of apoptotic process” including cysteine endopeptidases, “regulation of MAPK cascade” and “Pathways in cancer” (KEGG:05200). Interestingly, we found “Hematopoietic cell lineage” (KEGG:04640) and GO biological processes related to the immune system and inflammation such as “cytokine production (GO:0001816)” and “myeloid leukocyte activation” (Figure 4A). To facilitate data interpretation and reduce pathways redundancy among different ontologies, we converted the top 20 clusters of the enriched terms into enrichment network (Figure 4B). In the enrichment network, each term is represented by a circle node, whose size is proportional to the number of input genes that fall into that term, and its colour represents its cluster identity. Clusters with the most similar gene memberships are linked by an edge. Network visualization enables us to capture intra–cluster and inter–cluster similarities. Interestingly, as shown by the thickness of the edge (kappa similarity score), the closest relationships resulted among terms belonging to the immune system activation such as “Regulation of Cell Activation” (red), “myeloid leukocyte activation” (light purple), “regulation of binding” (white) and “Hematopoietic cell lineage” (light blue). Another group of related terms deals with blood coagulation and angiogenic processes such as “haemostasis” (pink), “chemotaxis” (grey) and “blood vessel development” (brown). We obtained similar enrichment results using G: profiler tool.

#### 2.4.2. lncRNA–mRNA Co–Expression Network

Functional analyses provided PCGs associated with biological processes in which also lncRNAs may be potentially involved. For this purpose, we performed Spearman’s rank correlation for the integrated dataset (Figure 1), i.e., the 30 candidate lncRNAs and 754 PCGs, in order to find the co–expression lncRNA–mRNA pairs and then construct co–expression networks. As result, we found significant perfect correlations (correlation equal to 1) for all 30 lncRNAs; each lncRNA has at least three co-expressed mRNAs (out of the 754 PCGs), all lncRNA-mRNA pairs are not overlapping based on their genomic positions; and groups of lncRNAs shares the same co-expressed mRNAs (Table S1).

#### 2.4.3. Ex–Vivo Validation

**Figure 5:**
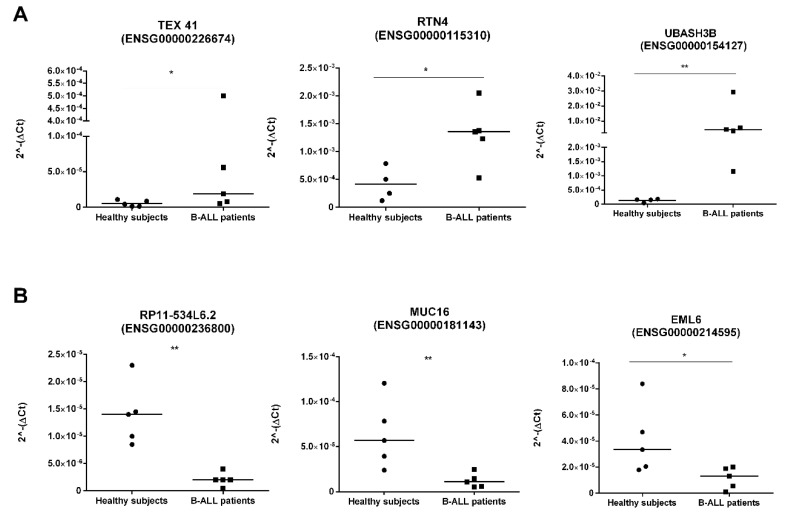
Expression level of the indicated lncRNA–mRNA pairs in healthy naive B cells (*n* = 5) and leukemic cells derived from paediatric B–ALL patients (*n* = 5). (**A**) lncRNA TEX41 and its co–expressed mRNAs (RTN4 and UBASH3B). (**B**) lncRNA RP11_534L62 and its co–expressed mRNAs (MUC16 and EML6). The relative expression level was determined using the 2^−ΔCt^ method and shown as median +/− SD of two technical independent experiments. * = *p*–value < 0.05; ** *p*–value < 0.01 unpaired–Mann–Whitney *t*–test.

We biologically validated lncRNA–mRNA pairs emerged from the co–expression network based on their higher expression changes (Table 1 and Table S1). We performed the RT–qPCR on the top–up–regulated lncRNA *TEX41* (log**_2_**FC 5.2, ENSG00000226674) and its two out of three co–expressed mRNAs such as *RTN4* (log**_2_**FC 1.8, ENSG00000115310) and *UBASH3B* (log**_2_**FC 4.4, ENSG00000154127) (Figure 5A), as well as the top down–regulated lncRNA *RP11_534L6.2* (log**_2_**FC−4.3, ENSG00000236800) and its two out of the thirty co–expressed mRNAs such as *MUC16*, (log**_2_**FC −5, ENSG00000181143) and *EML6* (log**_2_**FC –3.5, ENSG00000214595) (Figure 5B).

As expected, RT–qPCR experiments showed that the indicated lncRNA–mRNA pairs in perfect correlation have the same expression trend (down–regulation or up–regulation) across human B–ALL patients compared with healthy subjects. These results are in agreement with our in–silico analyses.

#### 2.4.4. KEGG Analysis of B–ALL Disease Associated Pathways

**Figure 6:**
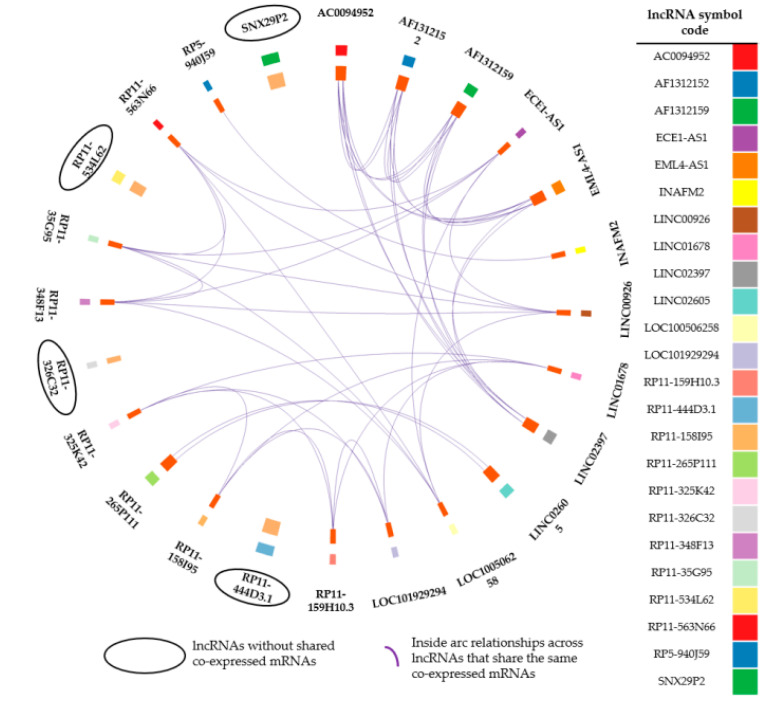
Circos plot shows overlap by lncRNAs and co–expressed mRNA lists of the 4 KEGG disease associated pathways. Circos outside reports lncRNA name, each one associated with its table colour. Circos inside arc represents for each lncRNA, mRNA memberships. Purple lines link the same gene that is shared by multiple gene lists (overlap). Dark orange colour represents the genes that appear in multiple lists and light orange colour represents genes that are unique to that gene list. A greater number of purple links and longer dark orange arcs implies greater overlap among lncRNA–mRNA lists.

In order to select the most relevant lncRNA–mRNA pairs underlying B–ALL disease phenotype, we chose from functional analyses the KEGG enriched terms related to tumorigenesis and leukaemia. This resulted in a total of 4 crucial pathways being detected, that included “Pathways in cancer” (Padj = 1.49 × 10^−4^), “Hematopoietic lineage” (Padj = 1.49 × 10^−4^), “Chronic myeloid leukaemia” (Padj = 4.29 × 10^−2^), “Acute myeloid leukaemia” (Padj = 4.29 × 10^−2^) (Table S2). Among the 30 lncRNAs, 24 resulted co–expressed with at least one PCG membership of the 4 KEGG pathways; several lncRNAs are pathway–specific, whereas other ones are shared between two or more pathways, indeed we distinguished five clusters, in which different lncRNAs share the same co–expressed mRNAs (Table S2). To highlight multiple layers of transcriptional regulation, i.e., lncRNAs–mRNAs, we performed a Circos plot (Figure 6).

Circos allows to explore simultaneously potential lncRNA–lncRNA and lncRNA–mRNA cooperation on the four KEGG disease associated pathways, according to the previous results. Circos outside represents the 24 lncRNAs with colour code legend. Circos inside bar size incorporates the number of co–expressed mRNAs for each lncRNA with bar colour indicating the presence of overlapping co–expressed mRNAs (dark orange bar), or the absence of overlapping mRNAs (light orange bar) among different lncRNAs. Circos inside arc (purple link) highlights the lncRNAs with overlapping co–expressed mRNAs (Figure 6).

In this way, we identified lncRNAs without arcs (*SNX29P2*; *RP11–444D3.1*; *RP11–326C3.2*; *RP11–534L62*) that could potentially act alone on their respectively co–expressed mRNAs, and lncRNAs with purple arcs acting potentially in concert on their shared co–expressed mRNAs (lncRNAs grouped in five distinct clusters in Table S2).

**Figure 7:**
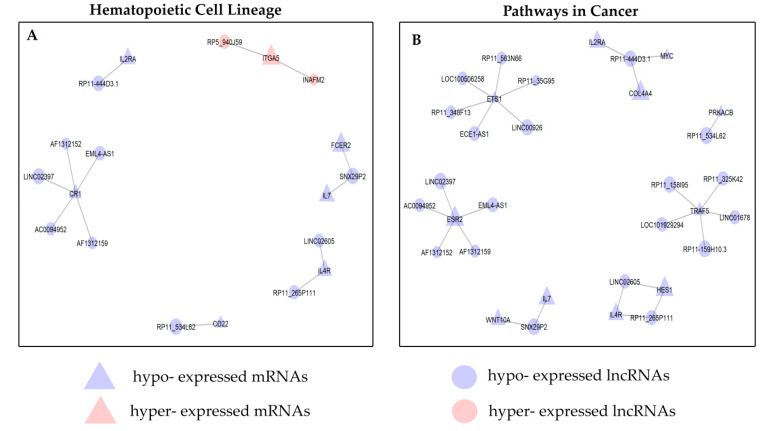
lncRNA–mRNA co–expression network. Network visualization of the KEGG enriched terms (**A**) “Hematopoietic cell lineage pathway” and (**B**) “Pathways in cancer”. The triangle and circular nodes represent mRNAs and lncRNAs, respectively. Red nodes represent increased expression comparing to healthy subjects and blue nodes represent decreased expression comparing to healthy subjects. lncRNA, long non–coding RNA.

Interestingly, about 80% of lncRNAs (24 out of 30) impact “Hematopoietic Cell Lineage” and “Pathways in cancer” signalling pathways and have at least one mRNA membership in these biological processes. For this reason, we constructed the co–expression networks underlying these two pathways (Figure 7).

The “Hematopoietic Cell Lineage” network is composed of a total of 19 nodes, of which 12 lncRNAs and 7 mRNAs linked by 13 edges. Interestingly, most modules included down–regulated genes, only one module include up–regulated genes (Figure 7A). The “Pathways in Cancer” network comprised 32 nodes, of which 21 lncRNAs and 11 mRNAs linked by 26 edges. All modules included down–regulated genes (Figure 7B). In “Chronic myeloid leukaemia”, we found that two lncRNAs (*RP11–444D3.1* and *RP11_326C3.2*) (Table S2) are co–expressed with two different mRNAs (*MYC* and *GAB2*, respectively). The pair *RP11–444D3.1–MYC* is co–expressed also in the “Acute myeloid leukaemia” (Table S2).

#### 2.4.5. Prediction of lncRNA Functions

Since lncRNAs’ functions are largely unknown, prediction of their functions may rely on the co–expression network using the guilt–by–association approach [28,29]. Among the 24 lncRNAs resulted involved in the four KEGG pathways (Table S2), we found that mRNA “partners” or “target” of some lncRNAs (*SNX29P2*, *LINC02605*, *RP11_265P111*, *RP11_534L6.2*, *RP11–444D3.1*, *AF1312159*, *EML4–AS1*, *AF1312152*, *AC009495.2*, *LINC02397*) enriched in GO biological process terms associated with cell communication, regulation of signal transduction and response to stimuli. Moreover, the mRNAs co–expressed with the lncRNAs *SNX29P2*, *LINC02605*, *RP11_265P111*, *AF1312159*, *EML4–AS1*, *AF1312152*, *AC009495.2* and *LINC02397* were enriched in GO biological process terms associated with to metabolic processes and in addition those ones co–expressed with the lncRNAs *AF1312159*, *EML4–AS1*, *AF1312152*, *AC009495.2* and *LINC02397* regulated the RNA biosynthetic process, thus playing an important role in the modulation of gene expression. The mRNAs co–expressed with the lncRNAs *LINC02605, RP11_265P111* and *RP11_534L6.2* were involved in the regulation of immune response and, in addition, those ones co–expressed with the lncRNAs *LINC02605* and *RP11_265P111* are also involved in the regulation of leukocyte activation and leukocyte cell–cell adhesion. Moreover, mRNAs co–expressed with the lncRNAs *LINC02605* and *RP11_265P111* are also involved in the regulation of cell population proliferation, cell–cell adhesion, cell activation and anatomical structure morphogenesis. Other additional biological processes are the cellular component organization, typical of the mRNAs co–expressed with the lncRNAs *LINC02605*, *RP11_265P111*, *RP11_534L62*, *AF1312159*, *EML4–AS1*, *AF1312152*, *AC009495.2* and *LINC02397*, and the multicellular organism development, typical of the mRNAs co–expressed with the lncRNAs *LINC02605*, *RP11_265P111* and *RP11–444D3.1*.

## 3. Discussion

Long non–coding RNAs are largely recognized as important players in cancer disease [7,51], including haematological malignancies [16,52,53]. In addition, considering that lncRNA expression is tightly controlled and exhibits even higher cell specificity than proteins, lncRNAs are emerging as a promising class of biomarkers and/or potential therapeutic targets [54,55,56,57]. Indeed, several lncRNA signatures for different tumours such as large–B–cell lymphoma, ovarian cancer, breast cancer and other oncological diseases have been reported [12,13,14,37,58,59,60,61,62]. However, few B–ALL–related lncRNAs have been characterized by their functional roles [15,52] and the lncRNA’s involvement in B–ALL development and/or progression as well as how they may orchestrate the B–ALL transcriptome landscape along with the protein–coding genes (PCGs) is far to be fully elucidated. In this context, we attempted to fill this knowledge gap by exploiting the transcriptome dataset already published by our research group [33] (Bioproject: PRJNA601326) for evaluating possible functional cooperation of lncRNAs with mRNAs. Our in–silico and ex vivo study aims to prioritize lncRNA signature as diagnostic biomarkers for paediatric B–ALL. To pursue this aim, we applied a PCA–based unsupervised feature extraction (FE) approach to select the most informative lncRNAs based on their expression variance. The PCA method, exclusively performed on lncRNAs, enabled us to address the bias due to the lower lncRNA expression levels when compared with PCG. Moreover, by making an a priori normalization, to obtain homoscedastic lncRNA values, all lncRNA genes give a roughly equal contribution to PCs.

In this way, we reduced the dimensionality of our transcriptomic dataset by removing the redundant features. As consequence, we identified a set of lncRNAs showing significant differences in the expression levels between B–ALL patients and healthy subjects. Next, we constructed a co–expression network (based on Spearman’s correlation) of the aberrantly expressed lncRNAs and PCGs) to predict lncRNA functions based on guilt–by–association approach. Only the perfectly correlated (rho = 1 and *p*–value < 0.005) lncRNA–mRNA pairs were extracted from the correlation matrix and used for downstream analyses. Our approach is in agreement with the literature [28,29,30,31,32].

We identified PCA–based 584 lncRNAs that effectively discriminate healthy subjects from B–ALL patients (Figure 2). In addition, we found that n. 30 lncRNAs showed an aberrant expression pattern in B–ALL patients (Table 1). Recognizing the importance of validations, we carried out (i) in–silico validation using the Bioproject PRJNA475681 (REF) as the external dataset and (ii) ex vivo validation of the most dysregulated lncRNA–mRNA pairs by RT–qPCR. Childhood B–ALL by Black et al., 2018 by [50] results are in agreement with our findings (Figure 3); indeed, 25 out of the 30 candidate lncRNAs showed expression trend concordance (up–regulated or down–regulated) with our RNA–Seq data (Bioproject PRJNA601326). Moreover, the ex–vivo RT–qPCR experiments on lncRNA–mRNA pairs, either in perfect correlation and with the highest (absolute value) fold change, also supported our RNA–Seq in–silico findings (Figure 5).

After checking that the genomic positions of the lncRNA–mRNA pairs in perfect correlation were not overlapping (Table S1), we give deep insight into lncRNA functional implications in B–ALL.

To simplify the inference of unknown lncRNA functions, we applied the “guilt–by–association” approach, using lncRNA co–expressed protein–coding genes (PCGs). The idea is that genes within a co–expressed module frequently may contribute to the same or similar functions tending to be involved in the same pathway. This approach has already proved efficacy to discover regulatory networks for different cancer [28,29,30,31,32]. In the field of leukaemia, Luo et al. [3] designed a mRNA–miRNA co–expression network for paediatric T cell acute lymphoblastic leukaemia (T–ALL); Pan et al. [63] constructed a lncRNA–lncRNA network for B cell acute myeloid leukaemia; Chen et al. [64] found survival specific regulatory network for lncRNAs and mRNAs in adult acute myeloid leukaemia; Zhang et al. [65] constructed a gene co–expression network for chronic lymphocytic leukaemia (CLL). To the best of our knowledge, the PCA–based unsupervised feature extraction (FE) approach followed by a co–expression network analysis for finding lncRNA signatures, has never been applied on paediatric B cell acute lymphoblastic leukaemia.

Functional enrichment analyses on co–expressed PCGs (Figure 4 and Table S2) indicated four KEGG pathways potentially associated with B–ALL: “Pathways in Cancer”, “Hematopoietic Cell Lineage”, “Chronic myeloid leukaemia” and “Acute myeloid leukaemia”.

Among the 30 lncRNAs, 24 resulted co–expressed with at least one PCG membership of the four KEGG pathways. Noteworthy, we identified two regulatory patterns: (i) lncRNA acting as clusters and potentially regulating the same co–expressed mRNA “partner” or “target” and (ii) lncRNAs potentially acting alone on their co–expressed mRNAs (Figure 6 and Table S2).

The majority of the 24 lncRNAs are featured by previously uncharacterized functions, and only a few of them (*LINC02397*, *AC009495.2*, *RP11_326C3.2*, *LINC00926*, *LOC100506258*, *LINC00984*, *RP11–79H23.3*) have been reported in the literature for other cancer types. LINC02397 has been reported to be involved in metastatic melanoma [34]. *AC009495.2* suppresses tumour metastasis by activating the *HDAC8/ID2* pathway in hepatocellular carcinoma [40]. *RP11–326C3.2* is involved in breast cancer (ER/PR positive type) by interaction with RAS associated genes [48] and plays role during human erythroid differentiation [49]. *LINC0092*6 may be useful for predicting prognosis among patients with thymoma [11]. *CTD–2516F10.2* also known as *LOC100506258* resulted among the genetic loci associated with IgG glycosylation [41]. LINC00984 is expressed in the human cardiovascular system [46,47]. *RP11–79H23.3* suppresses the pathogenesis and development of the bladder cancer, acting as competing endogenous RNA to increase the phosphatase and tensin homolog (*PTEN*) expression [38,39].

According to the gene ontology (GO) biological processes of the perfectly correlated PCGs closely associated with B–ALL disease, lncRNAs putative functions might refer to cancer processes. In particular, the mRNA partners of our lncRNAs are involved in cell communication, cell proliferation, cell–cell activation, regulation of signal transduction, response to stimuli and regulation of immune response (regulation of leukocyte activation and of the leukocyte cell–cell adhesion). In addition, there are important literature findings supporting that the identified mRNAs are involved in different types of neoplasms. For example, the interleukin–7 (IL–7), *FCER2* and *HES1* transcription factor have been already demonstrated to be necessary for the proliferation and differentiation of lymphoid progenitors of B and T cells [66,67,68]. *WNT10A* and the serine/threonine kinase *PRKACB* are well known to be implicated in oncogenesis through the activation of *WNT*–beta–catenin and cAMP signalling pathway, respectively [69]. In addition, the proto–oncogene MYC [70] and interleukin 2 receptor subunit Alpha (*IL2RA*) [71] are implicated in the prognosis of acute lymphoblastic leukaemia.

However, this study has some limitations. The major limitation of this work is the dataset’s low sample size. Unfortunately, few public transcriptomic datasets are available on childhood B–ALL including also healthy subjects (Cuadros et al., 2019 [72]; Black et al., 2018 [50]; Lajoie et al., 2017 [20]). Most of them are not or only partially comparable with our dataset due to (i) different cytogenetics of B–ALL samples; (ii) different high throughput platforms. Nevertheless, we performed the validation on the Black et al. (PRJNA475681) dataset, in particular using their 3 B–ALL patients with t(12;21) (ETV6/RUNX1) and their healthy subjects. The in–silico and ex vivo validations confirm the expression trend of lncRNAs and co–expressed mRNAs, thus gaining higher confidence in our preliminary data and encouraging for further analysis on a larger cohort. In addition, although we captured co–expression lncRNA–mRNA pairs from RNA–Seq data, it is well–established that co–expression correlation does not imply causation. Thus, gene perturbation experimental data would be necessary to gain insight on possible regulatory relationships or infer causal gene co–expression patterns [73].

Overall, our results suggest that the herein identified dysregulated lncRNAs by cooperating with cancer–related mRNAs may be potentially involved in the B–ALL disease. In the future, more mechanistic investigations should be warranted to better understand their regulatory network in B–ALL as well as further investigations to unveil their diagnostic and therapeutic potential in B–ALL patients management.

## 4. Materials and Methods

### 4.1. Identification of Differentially Expressed mRNAs and lncRNAs in B–ALL

Expression profiles of healthy circulating naive B cells and leukemic cells of children affected by common B–cells of children acute lymphoid leukaemia (B–ALL) are described in [33]. RNA–Seq fastq files are available at Bioproject PRJNA601326. Briefly, raw read counts were filtered for non–zero counts and normalized by applying the upper quartile (UQUA) approach. Differentially expressed genes (DEGs) between healthy subjects and tumour patients were defined as those genes having |log_2_FC| ≥  1.5, padj ≤ 0.05, computed as previously described [33]. Genes were annotated with Ensembl BioMart databank GRCh37.p13 version and biomarker applications were retrieved by Ingenuity Pathway Analysis (IPA) software (QIAGEN Inc., https://www.qiagenbioinformatics.com/products/ingenuitypathway-analysis) [74].

### 4.2. Gene Selection Using PCA–Based Unsupervised Feature Extraction (FE) Approach

To identify lncRNAs able to discriminate healthy subjects from B–ALL patients, we performed a PCA–based unsupervised feature extraction (FE) analysis, using as input the variance stabilized expression levels of lncRNAs regardless by their differential status. Variance stabilizing transformation (vst) was performed with DESeq2 R package [75] and was applied to raw count data to account for the different variances for the individual genes. After computing the correlation matrix of the d–dimensional dataset, this matrix was decomposed into its eigenvectors and eigenvalues. Eigenvectors were sorted by decreasing order based on their eigenvalues and then we selected the k eigenvectors corresponding to the k largest eigenvalues, where k is the dimensionality of the new feature subspace (k << d). This allowed us to extract only the most informative feature vectors, that is the eigenvectors with highest variance. The first principal component has the largest possible variance and all the consequent principal components have the largest variance given the constraint that these components are uncorrelated (orthogonal) to the other principal components. Finally, we selected the most significantly associated variables (lncRNAs) with each principal component (Pearson’s correlation *r* ≥ 0.8, *p*–value < 0.05)

Then, the previously identified differentially expressed lncRNAs were used to further filter the lncRNAs extracted within the PCA analysis.

### 4.3. In–Silico Validation

Results were compared to public childhood B–ALL RNA–Seq dataset (Accession Number PRJNA475681, by Black et al., 2018 [50]) using the normalized counts available for B–ALL patients with t(12;21) (ETV6/RUNX1) (Accession Number GSE115653) versus healthy subjects (Accession Number GSE1156555). We evaluated the expression trend in terms of log_2_FC comparing the public and internal dataset.

### 4.4. Ex Vivo Validation

RNA from primary bone marrow mononuclear cells (BM–MNC) of B–ALL patients and from B–cells purified (#19054, Easy Sep Human B Cell Enrichment kit, Stemcell Thecnologies, Vancouver, Canada) from healthy subjects were isolated using Trizol Reagent protocol (Thermo Fischer Scientific, Waltham, Massachusetts, MA, USA). The total RNA extracted was evaluated using QubitTM 4 Fluorometer (Thermo Fischer Scientific). cDNAs syntheses were performed using SuperScriptTM III First–Strand Synthesis SuperMix kit (Thermo Fisher Scientific) according to the manufacturer’s instructions. *RPS18* gene was used as housekeeping.

Oligonucleotides used for RT–qPCR were (Table 2):

RT–qPCR experiments were performed using C1000 Touch Thermal Cycler (Bio–Rad, Hercules, California, CA, USA) using iQ SYBR Green Supermix (#1708882, Bio–Rad). The following thermal protocol has been applied: initial denaturation (95 °C, 3  min), 40 cycles of denaturation (95 °C, 10 s), annealing (60 °C, 30 s) and elongation (72 °C, 30 s), final elongation (72 °C, 10 min) and a final hold (4 °C). The melting curve was generated in the range of 60–95 °C. The reaction volume was 1 µL. Each reaction was performed in duplicate. Samples were normalized to their RPS18 level using the 2−Δ*C*t method. Two independent experiments were performed for each RT–qPCR. Data were analysed using Biorad CFX Maestro version 1.0 (Bio–Rad).

### 4.5. Functional Enrichment Analysis and Guilt–By–Association Approach

The guilt–by–association approach was used to predict biological processes of novel lncRNAs. Functional enrichment analysis was performed with Metascape [76] and G:profiler [77] version e98_eg45_p14_ce5b097 on 754 protein–coding genes (PCGs) from RNA–Seq data [33]. The Metascape enrichment terms were filtered for −log10(*p*) > 6, cumulative hypergeometric *p*–values. The top 20 clusters from multiple ontologies were shown as bar graph and enrichment network based on Kappa–statistical similarities (score > 0.3) to dissect intra–cluster and inter–cluster similarities. In the G:profiler analysis we set up the statistical significance threshold at 0.05 (Benjamini–Hochberg FDR correction). Enrichment analysis, network and Circos have been generated by Metascape Cytoscape (v3.1.2) plug in. Both databases not included in the enrichment output the uncharacterized protein C12orf74 (ENSG00000214215).

### 4.6. Co–Expression Network Construction

Co–expression network analysis was performed by using the LINC R package [78]. Pairwise Spearman’s rank correlations (without other statistical corrections) were computed between the upper quantile–normalized expression values for each lncRNA–mRNA pair. To achieve a strong statistical robustness of correlation, only the co–expressed lncRNA–mRNA pairs with rho = 1 and *p*–value < 0.005 were included in the co–expression network. By applying this cut–off, we retained only gene pairs co–expressed in the same direction (i.e., positively or negatively). The network was then visualized using the GGNET R package [79].

### 4.7. Statistical Analysis

All statistical analyses and plots were performed using the open source R software (R version 3.5.2, https://www.R-project.org/) [80]. For statistical significance, a *p*–value less than 0.05 was considered, unless otherwise specified.

## 5. Conclusions

In the last years, lncRNAs have been implicated in many cancer types as diagnostic, prognostic or therapeutic targets. Moreover, little is known about the functional impact of lncRNAs in acute lymphoblastic leukaemia (ALL) aetiology, progression and treatment response [55]. Acute lymphoblastic leukaemia involves abnormal proliferation and differentiation of precursor lymphoid cell. Diagnosis incidence peak of ALL appears approximatively between 1 and 19 years old, as pre–B–ALL, pre–B–ALL with recurrent cytogenetic aberrations or precursor T–ALL, associated with diverse responsiveness to treatments and clinical outcome [2,3,4,5]. Traditionally, risk stratification of B–ALL encompasses clinical–pathological factors (age, white blood cell count), immunophenotype and response to chemotherapy [1]. In the recent years, several lncRNAs have been associated with leukaemia both on a diagnostic [19] and prognostic level to evaluate the patient response to treatment with prednisone [18,20].

In this study, we used next–generation sequencing (NGS), PCA and co–expression network approach, to discover lncRNAs strongly correlated with aberrantly expressed protein–coding genes underlying B–ALL pathogenesis. These lncRNAs deserve future investigations as potential diagnostic and therapeutic targets to yield more precise medicine in B–ALL patient management [5,6].

## Figures and Tables

**Table 1:** Annotations and expression profiling of the 30 lncRNA signatures as diagnostic candidates in paediatric B–ALL.

Ensembl ID	LncRNA Symbol GhR37 p.13/GhR38 p.13	Log_2_FC ^a^	*p* Adjusted	Average Expression Healthy Subjects	Average Expression Tumour Samples	Expression Trend	References
ENSG00000236800	*RP11–534L6.2/AC068898.1*	−4.368	3.05 × 10^−3^	15.21	0.15	Down–regulated	none
ENSG00000198106	*SNX29P2/AC025279.1*	−4.203	8.97 × 10^−8^	149.57	6.56	Down–regulated	none
ENSG00000246528	*RP11–159H10.3/AC079089.1*	−4.182	2.01 × 10^−3^	18.54	0.32	Down–regulated	none
ENSG00000205056	*RP11–693J15.5/LINC02397*	−4.149	4.69 × 10^−6^	624.81	25.57	Down–regulated	[34]
ENSG00000245869	*RP11–158I9.5/AP004609.3*	−4.053	3.60 × 10^−5^	78.48	3.03	Down–regulated	none
ENSG00000251538	*RP11–166A12.1/LINC02201*	−3.989	9.89 × 10^−3^	11.20	0.14	Down–regulated	[35]
ENSG00000255864	*RP11–444D3.1/not annotated*	−3.961	7.50 × 10^−3^	108.99	11.97	Down–regulated	none
ENSG00000247982	*LINC00926/LINC00926*	−3.918	2.21 × 10^−7^	4566.18	244.07	Down–regulated	[12]
ENSG00000237438	*CECR7/CECR7*	−3.691	6.59 × 10^−6^	105.06	6.23	Down–regulated	[36,37]
ENSG00000258810	*RP11–219E7.1/AL133371.2*	−3.399	6.77 × 10^−4^	37.33	2.64	Down–regulated	none
ENSG00000224875	*AC083949.1/EML4–AS1*	−3.369	1.21 × 10^−5^	50.18	4.33	Down–regulated	none
ENSG00000231105	*RP5–1071N3.1/ECE1–AS1*	−3.309	5.67 × 10^−7^	271.25	24.22	Down–regulated	none
ENSG00000224610	*RP11–265P11.1/AC108879.1*	−3.305	1.64 × 10^−3^	42.31	2.89	Down–regulated	none
ENSG00000253535	*RP11–624C23.1/AC120193.1*	−3.213	3.00 × 10^−4^	236.05	19.76	Down–regulated	[8]
ENSG00000261618	*RP11–79H23.3/LINC02605*	−3.13	2.16 × 10^−3^	38.87	3.35	Down–regulated	[38,39]
ENSG00000261114	*RP11–325K4.2/AC012181.1*	−3.112	1.40 × 10^−8^	142.67	14.54	Down–regulated	none
ENSG00000225331	*AP001055.6/LINC01678*	−3.054	5.02 × 10^−3^	27.10	2.35	Down–regulated	none
ENSG00000235192	*AC009495.2/AC009495.3*	−2.865	8.94 × 10^−3^	26.86	2.88	Down–regulated	[40]
ENSG00000251364	*CTD–2516F10.2/AC107884.1*	−2.827	1.94 × 10^−5^	181.05	23.66	Down–regulated	[41]
ENSG00000229151	*RP11–348F1.3/AC233976.1*	−2.755	6.32 × 10^−3^	34.97	4.25	Down–regulated	none
ENSG00000228403	*RP11–563N6.6/AC035139.1*	−2.557	2.74 × 10^−3^	46.95	7.03	Down–regulated	none
ENSG00000269918	*AF131215.9/AF131215.6*	−2.533	1.97 × 10^−3^	46.61	7.17	Down–regulated	none
ENSG00000255310	*AF131215.2/AF131215.5*	−2.236	4.47 × 10^−4^	97.41	19.14	Down–regulated	none
ENSG00000267787	*RP11–35G9.5/AC027097.2*	−1.86	1.21 × 10^−3^	216.93	57.25	Down–regulated	none
ENSG00000226674	*TEX41/TEX41*	5.218	4.88 × 10^−7^	2.44	170.21	Up–regulated	[42,43,44]
ENSG00000100181	*TPTEP1/TPTEP1*	3.527	5.58 × 10^−4^	7.33	112.53	Up–regulated	[45,46]
ENSG00000269968	*RP5–940J5.9/AC006064.4*	3.362	1.40 × 10^−4^	4.25	53.74	Up–regulated	none
ENSG00000259330	*LINC00984/INAFM2*	2.279	1.87 × 10^−4^	26.76	131.75	Up–regulated	[46,47]
ENSG00000224597	*PTCHD3P1/SVIL–AS1*	2.234	8.04 × 10^−4^	10.61	51.60	Up–regulated	[10]
ENSG00000255026	*RP11–326C3.2/AC136475.3*	2.211	3.15 × 10^−4^	31.38	147.22	Up–regulated	[48,49]

^a^ Differentially expressed lncRNAs (FC 1.5, Benjamini–Hochberg method, adjusted *p*-values < 0.05) ranked for log_2_ fold change (FC) according the expression trend.

**Table 2:** List of RT–qPCR oligonucleotides.

Gene	Forward Primer	Reverse Primer
RPS18:	fw 5′-CGATGGGCGGCGGAAAATA-3′;	rev 5′-CTGCTTTCCTCAACACCACA-3′;
TEX41:	fw 5′-TCATCTGTGAGGACCGTGAC-3′;	rev 5′-AGCACAGGAGAAGCTGAGTT-3′;
RTN4:	fw 5′-TGCGTCAGACTGTTCCACAC-3′;	rev 5′-CAGTCTCCTCTGCTGCACAA-3′;
UBASH3B:	fw 5′-GCTGGACGTGCTCCTCTC-3′;	rev 5′-AGTCACATGCTGCCTGAACA-3′;
CHST15:	fw 5′-CACACCAGATCCATCAAGGAC-3′;	rev 5′-TCCAGGCATTATTATCCCACA-3′;
RP11-534L6.2:	fw 5′-GGTGATGGTGATCAGGTGACT-3′;	rev 5′-TCGCTGCAGGGAGACTTC-3′;
MUC16:	fw 5′-CACAGTGGATGTGGGAACCT-3′;	rev 5′-GGTGAAGTTGAGGGTGAACG;
EML6:	fw 5′-GGGCAGGCAGAGGATTTC-3′;	rev 5′-TTTGGATCCGAAATTGACAGT-3′.

## Supplementary Materials

The following are available online at https://www.mdpi.com/2072-6694/12/9/2489/s1, Figure S1: lncRNAs expression level and library composition, Figure S2: Distribution of lncRNAs’ fold change (FC) among quartiles. lncRNAs extracted with PCA are reported in blue, while lncRNAs not extracted with PCA are reported in red. Figure S3: Gene expression profiling of 30 differential lncRNAs. Heatmap with hierarchical clustering shows the gene expression level, normalized by upper quartile (UQUA) approach, of 30 differential lncRNAs in B–ALL, Table S1: The genomic position of the (not overlapping) lncRNA–mRNA pairs, according to the GhCh37.p13. Following each lncRNA is reported its co–expressed mRNAs, Table S2: Functional roles of the 30 lncRNAs based on their perfect correlations (lncRNA–mRNAs) with mRNAs membership of the 4 KEGG disease associated pathways such as “Hematopoietic cell lineage “, “Pathways in cancer”, “Acute myeloid leukaemia, AML”, “Chronic myeloid leukaemia, CML”. Only lncRNAs grouped in clusters share the same co–expressed mRNAs.
