# Supplementary material for: lncRNAs–mRNAs Co–Expression Network Underlying Childhood B–Cell Acute Lymphoblastic Leukaemia: A Pilot Study
Source: Cancers (Basel). 2020 Sep 2;12(9):2489. doi: 10.3390/cancers12092489 (PMC7564554; doi:10.3390/cancers12092489)
Supplement: Supplementary file 1 [file cancers-12-02489-s001.pdf]

cancers

MDPI

Article

lncRNAs–mRNAs co–Expression Network Underlying Childhood B–Cell Acute Lymphoblastic Leukaemia: A Pilot Study

Ornella Affinito, Katia Pane, Giovanni Smaldone, Francesca Maria Orlandella, Peppino Mirabelli, Giuliana Beneduce, Rosanna Parasole, Mimmo Ripaldi, Marco Salvatore and Monica Franzese

Supplementary Materials

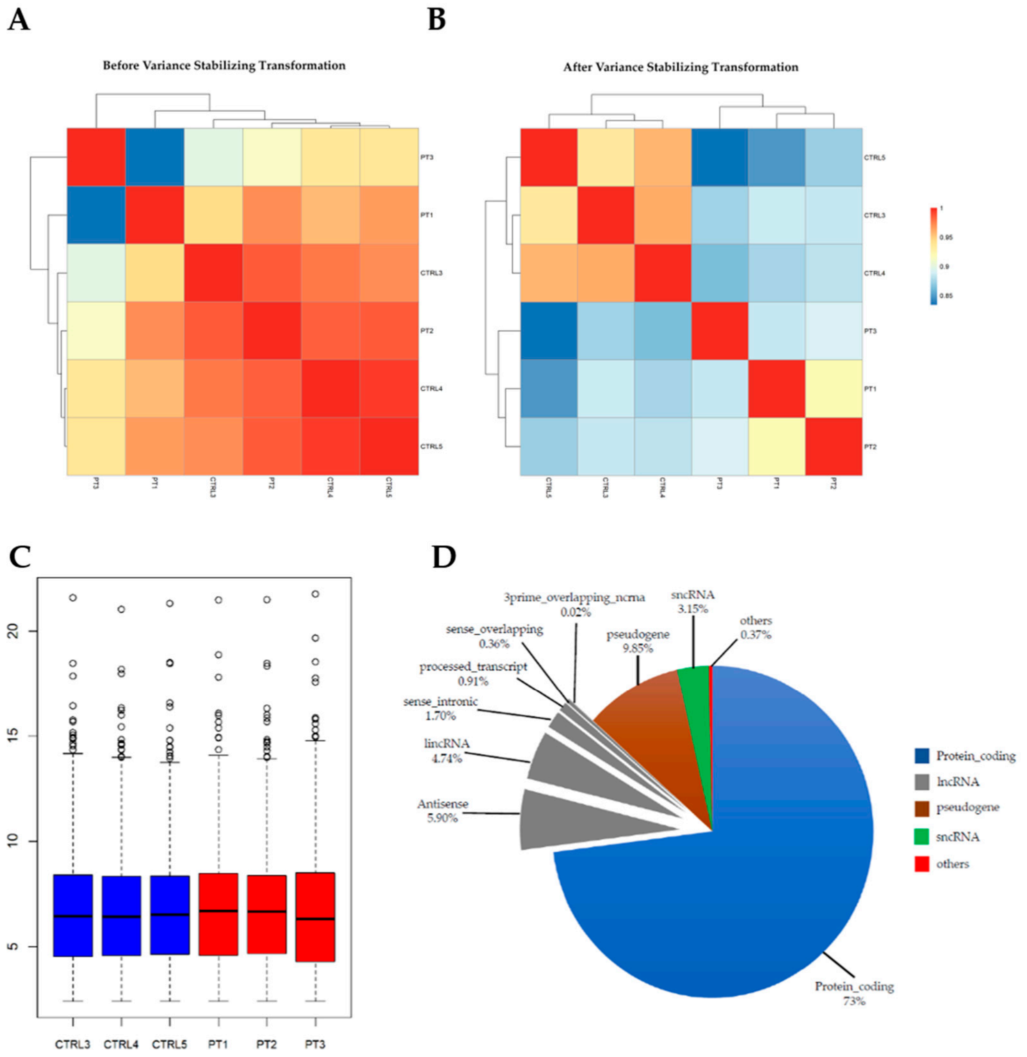

Figure S1. lncRNAs expression level and library composition. (**A**) Heatmap with hierarchical clustering using raw RNA expression levels and (**B**) normalized RNA–seq expression levels. Samples cluster together by sample group after variance stabilizing transformation. The colour scale indicates the degree of correlation (white–blue, low correlation; orange–red, strong correlation). (**C**) Boxplots show the distribution of expression levels after variance stabilizing transformation (vst) in each one of the samples. Healthy subjects are in blue and B–ALL patients are in red. (**D**) Pie chart of the distribution of detected genes into five summarized transcript types as indicated by Ensembl

BioMart databank GRCh37.p13, expressed in percentages. LncRNAs are further divided into the following classes: antisense, long intergenic ncRNAs (lncRNAs), processed transcripts, sense–intronic and sense–overlapping (sense) and 3′ overlapping non coding rna (ncrna).

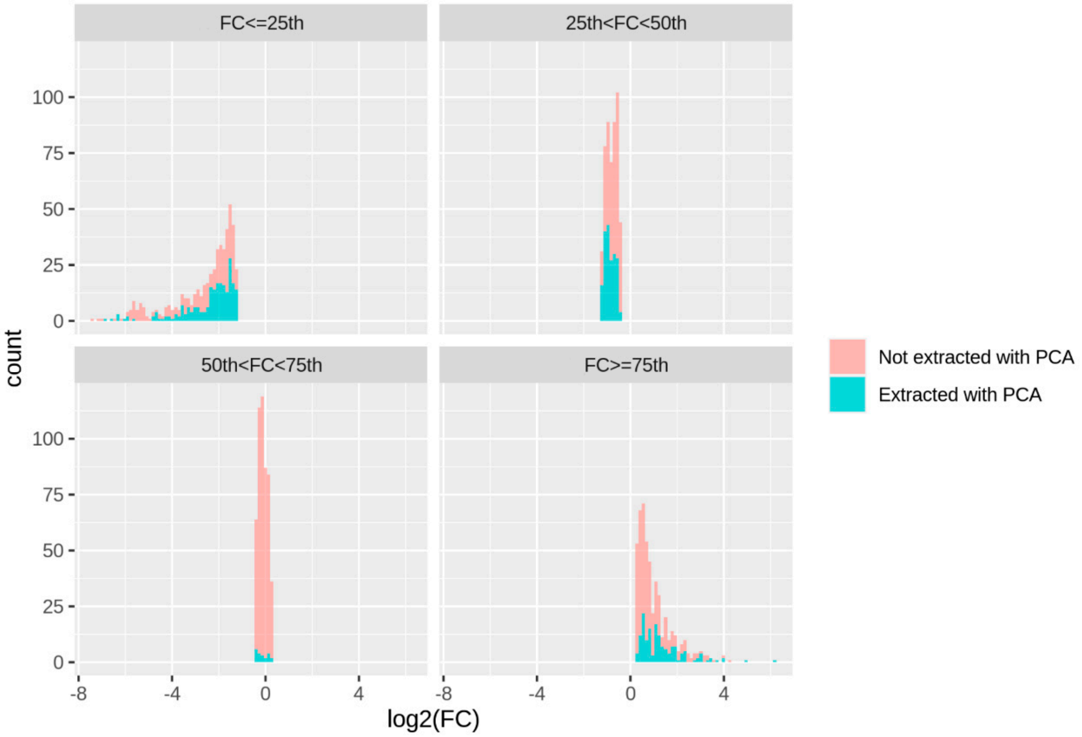

Figure S2. Distribution of lncRNAs fold change (FC) among quartiles. LncRNAs extracted with PCA are reported in blue, while lncRNAs not extracted with PCA are reported in red.

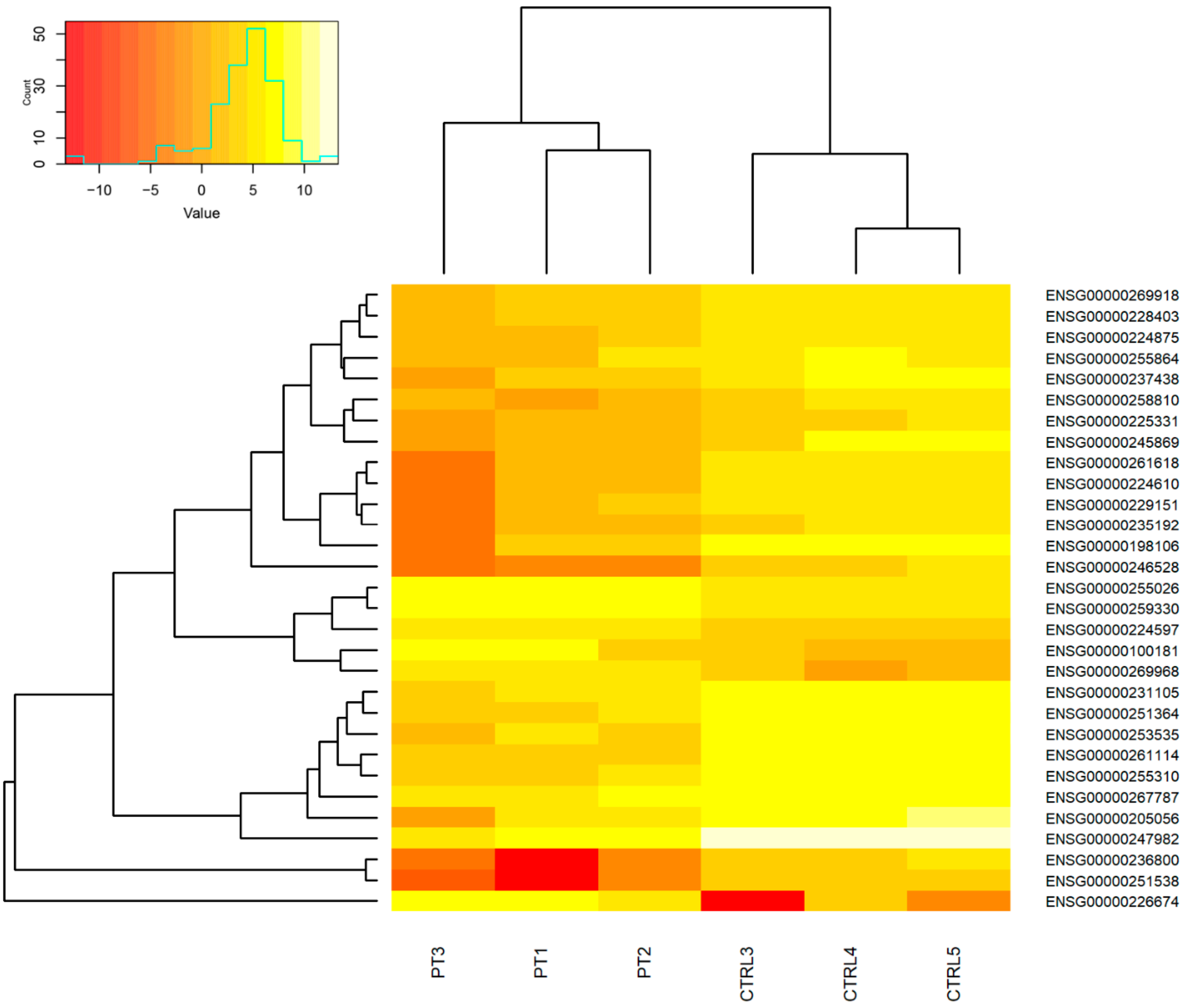

Figure S3. Gene expression profiling of 30 differential lncRNAs. Heatmap with hierarchical clustering shows the gene expression level, normalized by upper quartile (UQUA) approach, of 30 differential lncRNAs in B–ALL. Each column represents a subject, while each row represents the gene expression of a lncRNA. The color key indicates gene expression levels with red representing hypo–expression and yellow representing hyper–expression. The three B–ALL patients (PT1, PT2, and PT3) cluster together and are distinct from the 3 healthy subjects (CTRL3, CTRL4, CTRL5).

Table S1. The genomic position of the (not overlapping) lncRNA–mRNA pairs, according to the GhCh37.p13. Following each lncRNA or groups of lncRNAs are reported their co–expressed mRNAs.

Ensembl ID	Gene Name	Chromosome	Gene Start (bp)	Gene End (bp)	Gene Type	Correlation Direction
ENSG00000100181	*TPTEP1*	22	17082777	17179632	lincRNA	Up–regulated
ENSG00000067445	*TRO*	X	54946895	54957864	protein coding	Up–regulated
ENSG00000077097	*TOP2B*	3	25639475	25706398	protein coding	Up–regulated
ENSG00000109501	*WFS1*	4	6271576	6304992	protein coding	Up–regulated
ENSG00000119139	*TJP2*	9	71736209	71870124	protein coding	Up–regulated
ENSG00000125844	*RRBP1*	20	17594323	17662940	protein coding	Up–regulated
ENSG00000131773	*KHDRBS3*	8	136469700	136668965	protein coding	Up–regulated
ENSG00000136868	*SLC31A1*	9	115983808	116028674	protein coding	Up–regulated
ENSG00000198106	*SNX29P2*	16	29262829	29519817	lincRNA	Down–regulated
ENSG00000026559	*KCNG1*	20	49620193	49639666	protein coding	Down–regulated
ENSG00000082293	*COL19A1*	6	70576463	70919679	protein coding	Down–regulated
ENSG00000104432	*IL7*	8	79587978	79717758	protein coding	Down–regulated
ENSG00000104921	*FCER2*	19	7753644	7767032	protein coding	Down–regulated
ENSG00000105738	*SIPA1L3*	19	38397868	38699012	protein coding	Down–regulated
ENSG00000133985	*TTC9*	14	71108504	71142077	protein coding	Down–regulated
ENSG00000134242	*PTPN22*	1	114356433	114414381	protein coding	Down–regulated
ENSG00000135925	*WNT10A*	2	219745085	219764303	protein coding	Down–regulated
ENSG00000143297	*FCRL5*	1	157483167	157522310	protein coding	Down–regulated
ENSG00000147535	*PPAPDC1B*	8	38120648	38126761	protein coding	Down–regulated
ENSG00000149564	*ESAM*	11	124622026	124632186	protein coding	Down–regulated
ENSG00000154102	*C16orf74*	16	85723690	85784735	protein coding	Down–regulated
ENSG00000158050	*DUSP2*	2	96808905	96811179	protein coding	Down–regulated
ENSG00000163545	*NUAK2*	1	205271187	205290883	protein coding	Down–regulated
ENSG00000164649	*CDCA7L*	7	21940518	21985702	protein coding	Down–regulated
ENSG00000169507	*SLC38A11*	2	165752696	165812035	protein coding	Down–regulated
ENSG00000187912	*CLEC17A*	19	14693896	14721969	protein coding	Down–regulated
ENSG00000197705	*KLHL14*	18	30252634	30353025	protein coding	Down–regulated
ENSG00000269404	*SPIB*	19	50922195	50934570	protein coding	Down–regulated
ENSG00000205056	*RP11–693J15.5*	12	92860227	92885867	lincRNA	Down–regulated
ENSG00000224875	*AC083949.1*	2	42370378	42397441	antisense	Down–regulated
ENSG00000235192	*AC009495.2*	2	166651361	166666520	lincRNA	Down–regulated
ENSG00000255310	*AF131215.2*	8	10965298	10967236	sense_intronic	Down–regulated
ENSG00000269918	*AF131215.9*	8	10962201	10964214	sense_intronic	Down–regulated
ENSG00000002587	*HS3ST1*	4	11394774	11431389	protein coding	Down–regulated
ENSG00000028277	*POU2F2*	19	42590263	42700737	protein coding	Down–regulated
ENSG00000071575	*TRIB2*	2	12857015	12882860	protein coding	Down–regulated
ENSG00000079931	*MOXD1*	6	132617194	132722684	protein coding	Down–regulated
ENSG00000110315	*RNF141*	11	10533225	10562777	protein coding	Down–regulated
ENSG00000117009	*KMO*	1	241695434	241758944	protein coding	Down–regulated
ENSG00000124256	*ZBP1*	20	56178902	56195632	protein coding	Down–regulated
ENSG00000128268	*MGAT3*	22	39853349	39888199	protein coding	Down–regulated
ENSG00000132185	*FCRLA*	1	161676762	161684142	protein coding	Down–regulated

Ensembl ID	Gene Name	Chromosome	Gene Start (bp)	Gene End (bp)	Gene Type	Correlation Direction
ENSG00000132589	*FLOT2*	17	27206353	27224697	protein coding	Down–regulated
ENSG00000132704	*FCRL2*	1	157715523	157746922	protein coding	Down–regulated
ENSG00000134121	*CHL1*	3	238279	451090	protein coding	Down–regulated
ENSG00000136279	*DBNL*	7	44084239	44109055	protein coding	Down–regulated
ENSG00000140009	*ESR2*	14	64550950	64804830	protein coding	Down–regulated
ENSG00000146215	*CRIP3*	6	43267448	43276535	protein coding	Down–regulated
ENSG00000157470	*FAM81A*	15	59664892	59815748	protein coding	Down–regulated
ENSG00000161405	*IKZF3*	17	37921198	38020441	protein coding	Down–regulated
ENSG00000162144	*CYB561A3*	11	61116217	61129771	protein coding	Down–regulated
ENSG00000162894	*FAIM3*	1	207077731	207096592	protein coding	Down–regulated
ENSG00000163534	*FCRL1*	1	157764193	157789895	protein coding	Down–regulated
ENSG00000167723	*TRPV3*	17	3413796	3461289	protein coding	Down–regulated
ENSG00000173559	*NABP1*	2	192542794	192561385	protein coding	Down–regulated
ENSG00000182489	*XKRX*	X	100168431	100184422	protein coding	Down–regulated
ENSG00000182700	*IGIP*	5	139505521	139508391	protein coding	Down–regulated
ENSG00000186265	*BTLA*	3	112182815	112218408	protein coding	Down–regulated
ENSG00000197385	*ZNF860*	3	32023263	32033120	protein coding	Down–regulated
ENSG00000203710	*CR1*	1	207669492	207813992	protein coding	Down–regulated
ENSG00000204852	*TCTN1*	12	111051832	111087235	protein coding	Down–regulated
ENSG00000214900	*C14orf182*	14	50448430	50474238	protein coding	Down–regulated
ENSG00000248483	*POU5F2*	5	93070508	93077343	protein coding	Down–regulated
ENSG00000224597	*PTCHD3P1*	10	29698331	29776674	antisense	Down–regulated
ENSG00000226674	*TEX41*	2	145425534	145940216	lincRNA	Down–regulated
ENSG00000115310	*RTN4*	2	55199325	55339757	protein coding	Down–regulated
ENSG00000154127	*UBASH3B*	11	122526383	122685181	protein coding	Down–regulated
ENSG00000182022	*CHST15*	10	125767184	125853206	protein coding	Down–regulated
ENSG00000224610	*RP11–265P11.1*	X	39085356	39159040	lincRNA	Down–regulated
ENSG00000261618	*RP11–79H23.3*	8	79749764	79752757	lincRNA	Down–regulated
ENSG00000077238	*IL4R*	16	27324989	27376099	protein coding	Down–regulated
ENSG00000101017	*CD40*	20	44746911	44758502	protein coding	Down–regulated
ENSG00000109684	*CLNK*	4	10488019	10686489	protein coding	Down–regulated
ENSG00000114315	*HES1*	3	193853934	193856521	protein coding	Down–regulated
ENSG00000132849	*INADL*	1	62208149	62629592	protein coding	Down–regulated
ENSG00000139266	*MARCH9*	12	58148881	58154190	protein coding	Down–regulated
ENSG00000145685	*LHFPL2*	5	77781038	78065844	protein coding	Down–regulated
ENSG00000151320	*AKAP6*	14	32798479	33300567	protein coding	Down–regulated
ENSG00000155016	*CYP2U1*	4	108852525	108874613	protein coding	Down–regulated
ENSG00000169116	*PARM1*	4	75858305	75975325	protein coding	Down–regulated
ENSG00000169499	*PLEKHA2*	8	38758753	38831428	protein coding	Down–regulated
ENSG00000186815	*TPCN1*	12	113658855	113736390	protein coding	Down–regulated
ENSG00000205268	*PDE7A*	8	66629745	66754557	protein coding	Down–regulated
ENSG00000225331	*AP001055.6*	21	45578623	45579959	lincRNA	Down–regulated
ENSG00000245869	*RP11–158I9.5*	11	118756550	118758451	antisense	Down–regulated
ENSG00000246528	*RP11–159H10.3*	8	70746346	70767206	antisense	Down–regulated
ENSG00000253535	*RP11–624C23.1*	8	24153327	24769586	antisense	Down–regulated

Ensembl ID	Gene Name	Chromosome	Gene Start (bp)	Gene End (bp)	Gene Type	Correlation Direction
ENSG00000261114	*RP11–325K4.2*	16	56974940	56975638	sense_intronic	Down–regulated
ENSG00000082512	*TRAF5*	1	211499957	211548288	protein coding	Down–regulated
ENSG00000111846	*GCNT2*	6	10492456	10629601	protein coding	Down–regulated
ENSG00000133740	*E2F5*	8	86089460	86129387	protein coding	Down–regulated
ENSG00000138185	*ENTPD1*	10	97471536	97637023	protein coding	Down–regulated
ENSG00000138867	*GUCD1*	22	24936406	24951903	protein coding	Down–regulated
ENSG00000147548	*WHSC1L1*	8	38127215	38239790	protein coding	Down–regulated
ENSG00000157483	*MYO1E*	15	59427113	59665099	protein coding	Down–regulated
ENSG00000162066	*AMDHD2*	16	2570358	2581423	protein coding	Down–regulated
ENSG00000174607	*UGT8*	4	115519611	115599380	protein coding	Down–regulated
ENSG00000188177	*ZC3H6*	2	113033171	113097640	protein coding	Down–regulated
ENSG00000228403	*RP11–563N6.6*	10	50086067	50086694	sense_intronic	Down–regulated
ENSG00000229151	*RP11–348F1.3*	X	51099796	51139314	antisense	Down–regulated
ENSG00000231105	*RP5–1071N3.1*	1	21619783	21626267	antisense	Down–regulated
ENSG00000247982	*LINC00926*	15	57592563	57599959	lincRNA	Down–regulated
ENSG00000251364	*CTD–2516F10.2*	11	7448497	7533746	antisense	Down–regulated
ENSG00000267787	*RP11–35G9.5*	18	55306687	55405276	antisense	Down–regulated
ENSG00000066294	*CD84*	1	160510885	160549306	protein coding	Down–regulated
ENSG00000073849	*ST6GAL1*	3	186648274	186796341	protein coding	Down–regulated
ENSG00000074657	*ZNF532*	18	56529832	56653712	protein coding	Down–regulated
ENSG00000079263	*SP140*	2	231067826	231223762	protein coding	Down–regulated
ENSG00000100307	*CBX7*	22	39516172	39548679	protein coding	Down–regulated
ENSG00000112320	*SOBP*	6	107811162	107981357	protein coding	Down–regulated
ENSG00000123096	*SSPN*	12	26274924	26452223	protein coding	Down–regulated
ENSG00000134954	*ETS1*	11	128328656	128457453	protein coding	Down–regulated
ENSG00000143869	*GDF7*	2	20866424	20873418	protein coding	Down–regulated
ENSG00000145416	*MARCH1*	4	164445450	165305202	protein coding	Down–regulated
ENSG00000145779	*TNFAIP8*	5	118604387	118735383	protein coding	Down–regulated
ENSG00000147457	*CHMP7*	8	23101150	23119512	protein coding	Down–regulated
ENSG00000147912	*FBXO10*	9	37510889	37588871	protein coding	Down–regulated
ENSG00000160991	*ORAI2*	7	102073553	102097268	protein coding	Down–regulated
ENSG00000163568	*AIM2*	1	159032274	159116886	protein coding	Down–regulated
ENSG00000170006	*TMEM154*	4	153539784	153601317	protein coding	Down–regulated
ENSG00000172663	*TMEM134*	11	67231824	67236743	protein coding	Down–regulated
ENSG00000229474	*PATL2*	15	44957930	45003514	protein coding	Down–regulated
ENSG00000236800	*RP11–534L6.2*	10	49872244	49880469	lincRNA	Down–regulated
ENSG00000012124	*CD22*	19	35810164	35838258	protein coding	Down–regulated
ENSG00000054219	*LY75*	2	160628362	160761260	protein coding	Down–regulated
ENSG00000077232	*DNAJC10*	2	183580999	183659191	protein coding	Down–regulated
ENSG00000111696	*NT5DC3*	12	104164231	104234975	protein coding	Down–regulated
ENSG00000117090	*SLAMF1*	1	160577890	160617085	protein coding	Down–regulated
ENSG00000133789	*SWAP70*	11	9685624	9774538	protein coding	Down–regulated
ENSG00000134061	*CD180*	5	66478103	66492627	protein coding	Down–regulated
ENSG00000135074	*ADAM19*	5	156822542	157002783	protein coding	Down–regulated
ENSG00000142875	*PRKACB*	1	84543745	84704181	protein coding	Down–regulated

Ensembl ID	Gene Name	Chromosome	Gene Start (bp)	Gene End (bp)	Gene Type	Correlation Direction
ENSG00000154511	*FAM69A*	1	93307724	93427057	protein coding	Down–regulated
ENSG00000156711	*MAPK13*	6	36095586	36107842	protein coding	Down–regulated
ENSG00000160602	*NEK8*	17	27052915	27070473	protein coding	Down–regulated
ENSG00000163617	*KIAA1407*	3	113682984	113775460	protein coding	Down–regulated
ENSG00000163644	*PPM1K*	4	89178772	89205921	protein coding	Down–regulated
ENSG00000174123	*TLR10*	4	38773860	38784611	protein coding	Down–regulated
ENSG00000174130	*TLR6*	4	38825336	38858438	protein coding	Down–regulated
ENSG00000181143	*MUC16*	19	8959520	9092018	protein coding	Down–regulated
ENSG00000185404	*SP140L*	2	231191899	231268447	protein coding	Down–regulated
ENSG00000186088	*GSAP*	7	76940068	77045717	protein coding	Down–regulated
ENSG00000214595	*EML6*	2	54950636	55199157	protein coding	Down–regulated
ENSG00000235750	*KIAA0040*	1	175126123	175162135	protein coding	Down–regulated
ENSG00000237438	*CECR7*	22	17517460	17541715	lincRNA	Down–regulated
ENSG00000042980	*ADAM28*	8	24151553	24216531	protein coding	Down–regulated
ENSG00000082438	*COBLL1*	2	165510134	165700189	protein coding	Down–regulated
ENSG00000100580	*TMED8*	14	77801364	77843452	protein coding	Down–regulated
ENSG00000123095	*BHLHE41*	12	26272959	26278060	protein coding	Down–regulated
ENSG00000149292	*TTC12*	11	113185251	113254266	protein coding	Down–regulated
ENSG00000161929	*SCIMP*	17	5112256	5138155	protein coding	Down–regulated
ENSG00000165025	*SYK*	9	93564069	93660831	protein coding	Down–regulated
ENSG00000251538	*RP11–166A12.1*	5	121964647	122066380	lincRNA	Down–regulated
ENSG00000124357	*NAGK*	2	71291474	71306935	protein coding	Down–regulated
ENSG00000124374	*PAIP2B*	2	71409869	71454213	protein coding	Down–regulated
ENSG00000163683	*SMIM14*	4	39547950	39640710	protein coding	Down–regulated
ENSG00000185666	*SYN3*	22	32908539	33454358	protein coding	Down–regulated
ENSG00000255026	*RP11–326C3.2*	11	287305	288987	antisense	Up–regulated
ENSG00000018510	*AGPS*	2	178257372	178408564	protein coding	Up–regulated
ENSG00000033327	*GAB2*	11	77926343	78129394	protein coding	Up–regulated
ENSG00000052795	*FNIP2*	4	159690290	159829201	protein coding	Up–regulated
ENSG00000106069	*CHN2*	7	29161890	29553944	protein coding	Up–regulated
ENSG00000108219	*TSPAN14*	10	82213922	82292879	protein coding	Up–regulated
ENSG00000109113	*RAB34*	17	27041299	27045447	protein coding	Up–regulated
ENSG00000113749	*HRH2*	5	175085033	175113245	protein coding	Up–regulated
ENSG00000115183	*TANC1*	2	159825146	160089170	protein coding	Up–regulated
ENSG00000117016	*RIMS3*	1	41086351	41131329	protein coding	Up–regulated
ENSG00000135404	*CD63*	12	56119107	56123491	protein coding	Up–regulated
ENSG00000135821	*GLUL*	1	182350839	182361341	protein coding	Up–regulated
ENSG00000143847	*PPFIA4*	1	202995626	203047868	protein coding	Up–regulated
ENSG00000152495	*CAMK4*	5	110559351	110830584	protein coding	Up–regulated
ENSG00000165801	*ARHGEF40*	14	21538429	21558399	protein coding	Up–regulated
ENSG00000169136	*ATF5*	19	50431959	50437192	protein coding	Up–regulated
ENSG00000177602	*GSG2*	17	3627211	3630067	protein coding	Up–regulated
ENSG00000178404	*DDC8*	17	76866992	76899299	protein coding	Up–regulated
ENSG00000182253	*SYNM*	15	99638420	99675798	protein coding	Up–regulated
ENSG00000182718	*ANXA2*	15	60639333	60695082	protein coding	Up–regulated

Ensembl ID	Gene Name	Chromosome	Gene Start (bp)	Gene End (bp)	Gene Type	Correlation Direction
ENSG00000198648	*STK39*	2	168810530	169104651	protein coding	Up–regulated
ENSG00000255864	*RP11–444D3.1*	12	24366190	24715524	lincRNA	Down–regulated
ENSG00000075035	*WSCD2*	12	108523248	108644314	protein coding	Down–regulated
ENSG00000078589	*P2RY10*	X	78200829	78217451	protein coding	Down–regulated
ENSG00000080007	*DDX43*	6	74104471	74127292	protein coding	Down–regulated
ENSG00000081052	*COL4A4*	2	227867427	228028829	protein coding	Down–regulated
ENSG00000105492	*SIGLEC6*	19	52022779	52035110	protein coding	Down–regulated
ENSG00000115825	*PRKD3*	2	37477645	37551951	protein coding	Down–regulated
ENSG00000118432	*CNR1*	6	88849583	88876078	protein coding	Down–regulated
ENSG00000124429	*POF1B*	X	84532402	84634748	protein coding	Down–regulated
ENSG00000134460	*IL2RA*	10	6052652	6104288	protein coding	Down–regulated
ENSG00000134532	*SOX5*	12	23682440	24103966	protein coding	Down–regulated
ENSG00000136997	*MYC*	8	128747680	128753674	protein coding	Down–regulated
ENSG00000162551	*ALPL*	1	21835858	21904905	protein coding	Down–regulated
ENSG00000163219	*ARHGAP25*	2	68906733	69053965	protein coding	Down–regulated
ENSG00000163637	*PRICKLE2*	3	64079543	64431152	protein coding	Down–regulated
ENSG00000164574	*GALNT10*	5	153570290	153800544	protein coding	Down–regulated
ENSG00000165521	*EML5*	14	89078775	89259096	protein coding	Down–regulated
ENSG00000170456	*DENND5B*	12	31535157	31744031	protein coding	Down–regulated
ENSG00000177311	*ZBTB38*	3	141043055	141168634	protein coding	Down–regulated
ENSG00000182240	*BACE2*	21	42539728	42654445	protein coding	Down–regulated
ENSG00000183091	*NEB*	2	152341850	152591001	protein coding	Down–regulated
ENSG00000187510	*PLEKHG7*	12	93115281	93166231	protein coding	Down–regulated
ENSG00000258810	*RP11–219E7.1*	14	21252047	21252452	antisense	Down–regulated
ENSG00000035720	*STAP1*	4	68424446	68473055	protein coding	Down–regulated
ENSG00000122224	*LY9*	1	160765864	160798051	protein coding	Down–regulated
ENSG00000153814	*JAZF1*	7	27870192	28220362	protein coding	Down–regulated
ENSG00000172869	*DMXL1*	5	118373467	118584833	protein coding	Down–regulated
ENSG00000259330	*LINC00984*	15	40617417	40618916	antisense	Up–regulated
ENSG00000269968	*RP5–940J5.9*	12	6646960	6647536	antisense	Up–regulated
ENSG00000008256	*CYTH3*	7	6201407	6312275	protein coding	Up–regulated
ENSG00000065534	*MYLK*	3	123328896	123603178	protein coding	Up–regulated
ENSG00000115355	*CCDC88A*	2	55514978	55647057	protein coding	Up–regulated
ENSG00000122779	*TRIM24*	7	138145079	138274738	protein coding	Up–regulated
ENSG00000132846	*ZBED3*	5	76367897	76383148	protein coding	Up–regulated
ENSG00000137266	*SLC22A23*	6	3269196	3457256	protein coding	Up–regulated
ENSG00000140836	*ZFHX3*	16	72816784	73093597	protein coding	Up–regulated
ENSG00000161638	*ITGA5*	12	54789045	54813244	protein coding	Up–regulated
ENSG00000165512	*ZNF22*	10	45495923	45500774	protein coding	Up–regulated
ENSG00000168268	*NT5DC2*	3	52558386	52569070	protein coding	Up–regulated
ENSG00000170340	*B3GNT2*	2	62423248	62451866	protein coding	Up–regulated
ENSG00000178695	*KCTD12*	13	77454312	77460540	protein coding	Up–regulated
ENSG00000179820	*MYADM*	19	54369477	54379691	protein coding	Up–regulated
ENSG00000198369	*SPRED2*	2	65537985	65659771	protein coding	Up–regulated
ENSG00000206560	*ANKRD28*	3	15708743	15901278	protein coding	Up–regulated

Table S2. Functional roles of the 30 lncRNAs based on their perfect correlations (lncRNA–mRNA) with mRNAs membership of the 4 KEGG disease associated pathways such as "Hematopoietic cell lineage ", "Pathways in cancer", "Acute myeloid leukaemia, AML", "Chronic myeloid leukaemia, CML". Only lncRNAs grouped in clusters share the same co–expressed mRNAs.

Cluster	lncRNA symbol (GRCh37.p13)/(GRCh38.p13)	Ensembl ID	n° of co–expressed mRNAs	mRNAs membership of KEGG disease associated pathways[a] (gene symbol)
1	*LINC02397/LINC02397*	ENSG00000205056	30	1/30 Hematopoietic: ENSG00000203710 (*CR1*)| 1/30 Pathways in cancer: ENSG00000140009 (*ESR2*)|
	EML4–AS1/EML4–AS1	ENSG00000224875		
	AC0094952/AC009495.3	ENSG00000235192		
	AF1312152/AF131215.5	ENSG00000255310		
	AF1312159/AF131215.6	ENSG00000269918		
–	*RP11_534L62/AC068898.1*	ENSG00000236800	21	1/21 Hematopoietic: ENSG00000012124 (*CD22*)| 1/21 Pathways in cancer: ENSG00000142875 (*PRKACB*)|
–	*RP11–444D3.1*	ENSG00000255864	21	1/21 Hematopoietic: ENSG00000134460 (*IL2RA*)| 3/21 Pathways in cancer: ENSG00000142875 (*COL4A4*); ENSG00000134460 (*IL2RA*); ENSG00000136997 (*MYC*)| 1/21 AML: ENSG00000136997 (*MYC*)| 1/21 CML: ENSG00000136997 (*MYC*)|
–	*RP11_326C32/AC136475.3*	ENSG00000255026	20	1/20 CML: ENSG00000033327 (*GAB2*)|
–	*SNX29P2/AC025279.1*	ENSG00000198106	19	2/19 Hematopoietic: ENSG00000104432 (*IL7*); ENSG0000010492 (*FCER2*)| 2/19 Pathways in cancer: ENSG00000104432 (*IL7*); ENSG00000135925 (*WNT10A*)|
2	*RP11_563N66/AC035139.1*	ENSG00000228403	18	1/18 Pathways in cancer: ENSG00000134954 (*ETS1*)|
	RP11_348F13/AC233976.1	ENSG00000229151		
	ECE1–AS1/ECE1–AS1	ENSG00000231105		
	LINC00926/LINC00926	ENSG00000247982		
	LOC100506258/AC107884.1	ENSG00000251364		
	RP11_35G95/AC027097.2	ENSG00000267787		
3	*RP5_940J59/AC006064.4*	ENSG00000269968	15	1/15 Hematopoietic: ENSG00000161638 (*ITGA5*)|
	LINC00984/INAFM2	ENSG00000259330		
4	*RP11_265P111/AC108879.1*	ENSG00000224610	13	1/13 Hematopoietic: ENSG00000077238 (*IL4R*)| 2/13 Pathways in cancer: ENSG00000077238 (*IL4R*); ENSG00000114315 (*HES1*)|
	RP11–79H23.3/LINC02605	ENSG00000261618		
5	*AP001055.6/LINC01678*	ENSG00000225331	10	1/10 Pathways in cancer: ENSG00000082512 (*TRAF5*)|
	RP11–158I9.5/AP004609.3	ENSG00000245869		
	RP11–159H10.3/AC079089.1	ENSG00000246528		
	LOC101929294/AC120193.1	ENSG00000253535		
	RP11_325K42/AC012181.1	ENSG00000261114		
–	*TPTEP1/TPTEP1*	ENSG00000100181	7	none involved in Hematopoietic, Pathways in cancer, AML or CML
–	*CECR7/CECR7*	ENSG00000237438	7	none involved in Hematopoietic, Pathways in cancer, AML or CML
–	*RP11–166A12.1/LINC02201*	ENSG00000251538	4	none involved in Hematopoietic, Pathways in cancer, AML or CML
–	*RP11_219E71/ AL133371.2*	ENSG00000258810	4	none involved in Hematopoietic, Pathways in cancer, AML or CML

Cluster	lncRNA symbol (GRCh37.p13)/(GRCh38.p13)	Ensembl ID	n° of co–expressed mRNAs	mRNAs membership of KEGG disease associated pathways[a] (gene symbol)
–	*PTCHD3P1*/*SVIL–AS1*	ENSG00000224597	3	none involved in Hematopoietic, Pathways in cancer, AML or CML
–	*TEX41*/*TEX41*	ENSG00000226674	3	none involved in Hematopoietic, Pathways in cancer, AML or CML

[a] KEGG disease associated pathways term id KEGG:04640, KEGG:05200, KEGG:05221 and KEGG:05220 from the full G:profiler enrichment analysis, padj, Benjamini–Hochberg method FDR<0.05. See Materials and Methods.

© 2020 by the authors. Licensee MDPI, Basel, Switzerland. This article is an open access article distributed under the terms and conditions of the Creative Commons Attribution (CC BY) license (http://creativecommons.org/licenses/by/4.0/).
